# Supplementary figures and images for: Structural insights into regulation of CNNM-TRPM7 divalent cation uptake by the small GTPase ARL15
Source: eLife. 2023 Jul 14;12:e86129. doi: 10.7554/eLife.86129 (PMC10348743; doi:10.7554/eLife.86129)

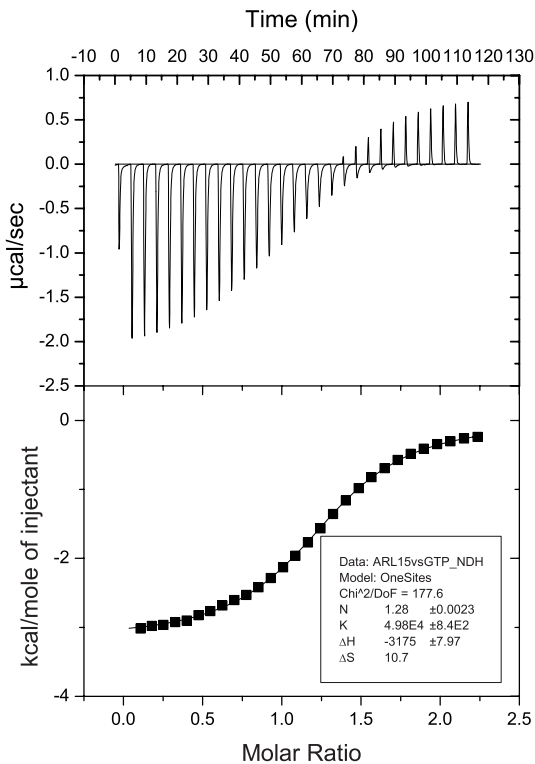

300  $\mu\text{M}$  ARL15 (32-197)  
+ 3 mM GTP

Supplement: Figure 1—source data 1. [file elife-86129-fig1-data1.pdf]

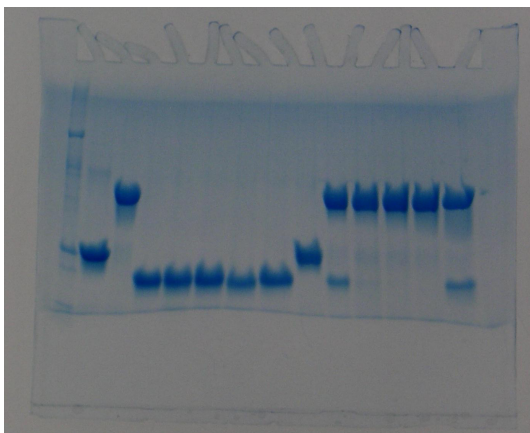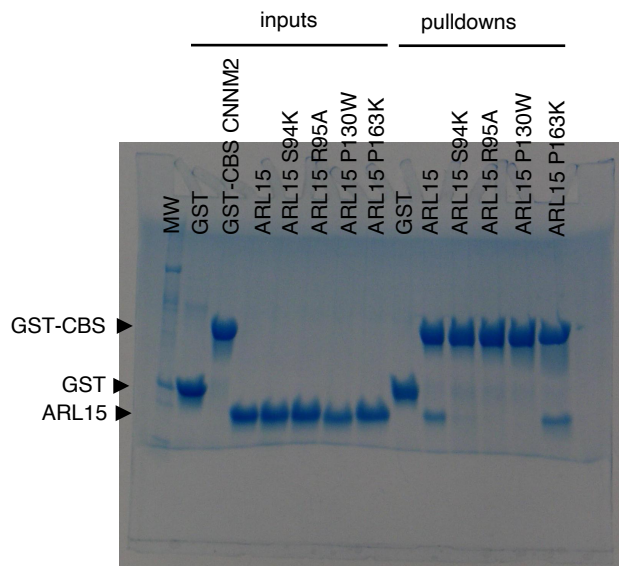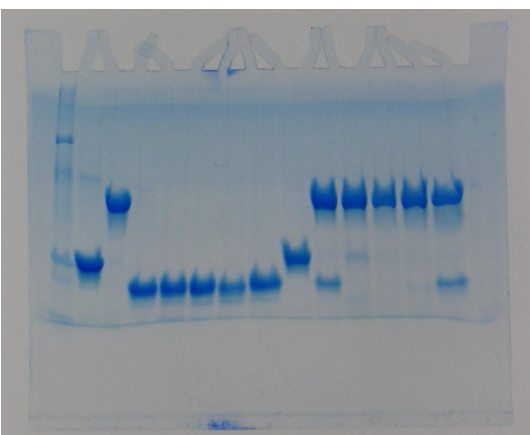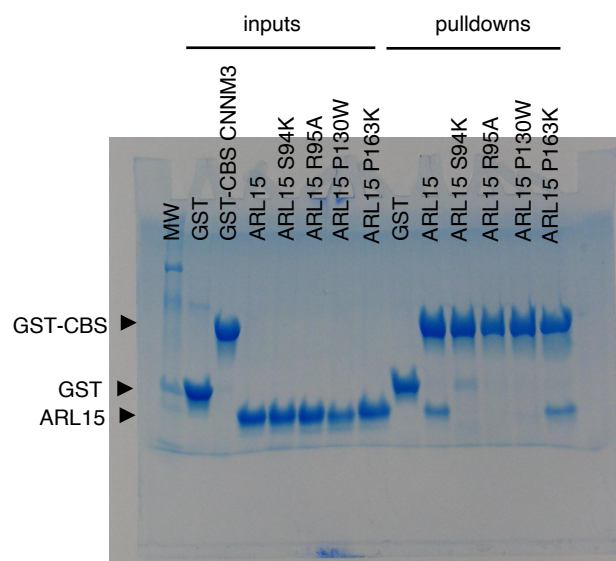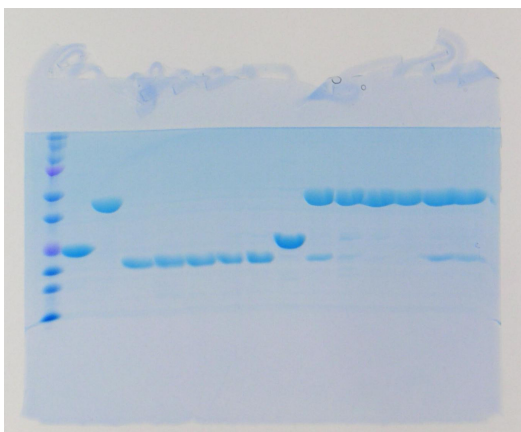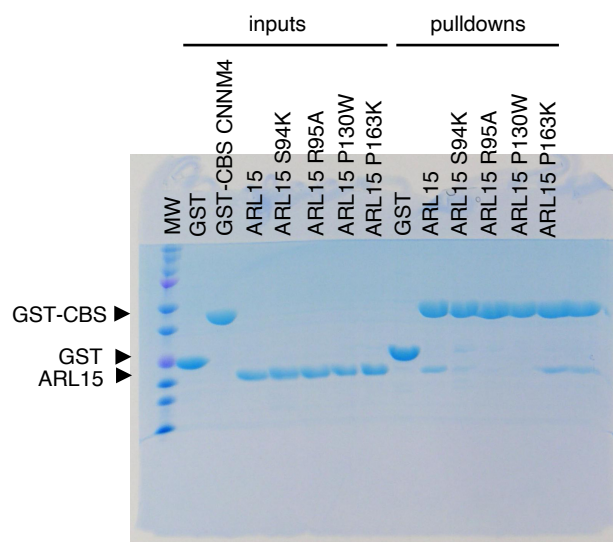

Supplement: Figure 4—source data 1. [file elife-86129-fig4-data1.pdf]

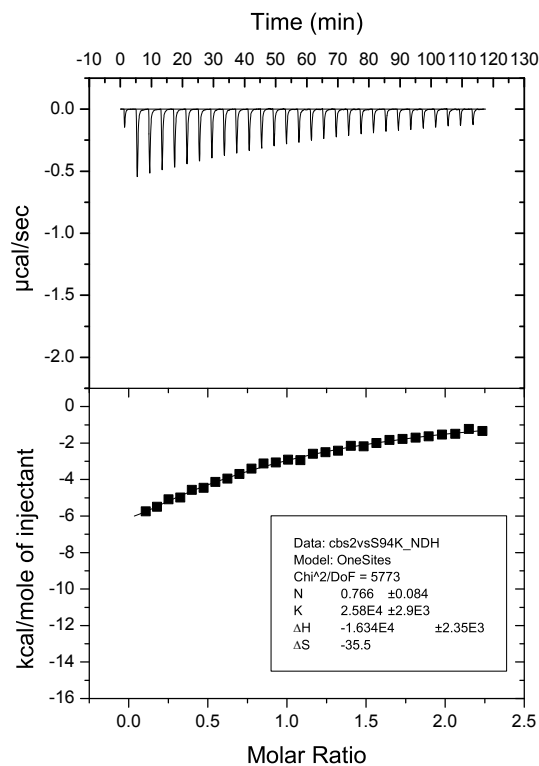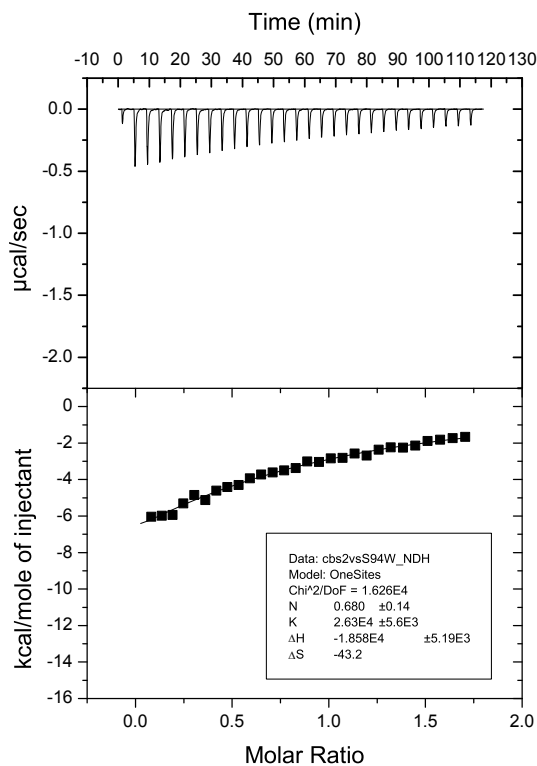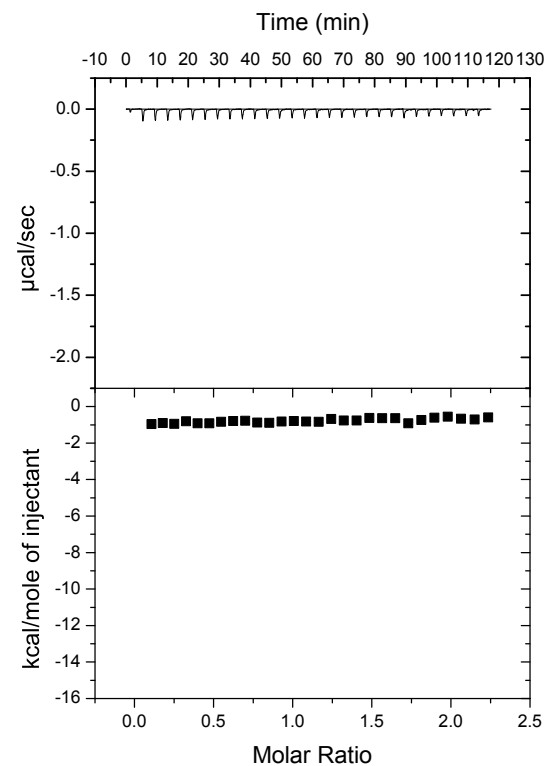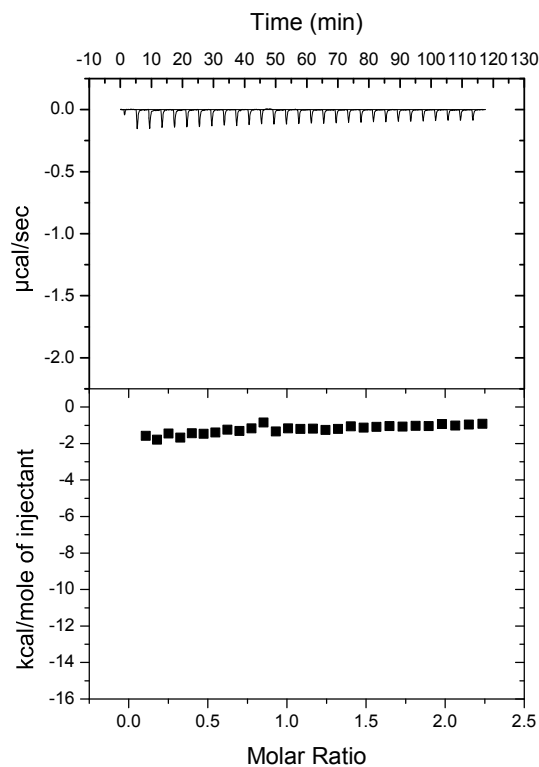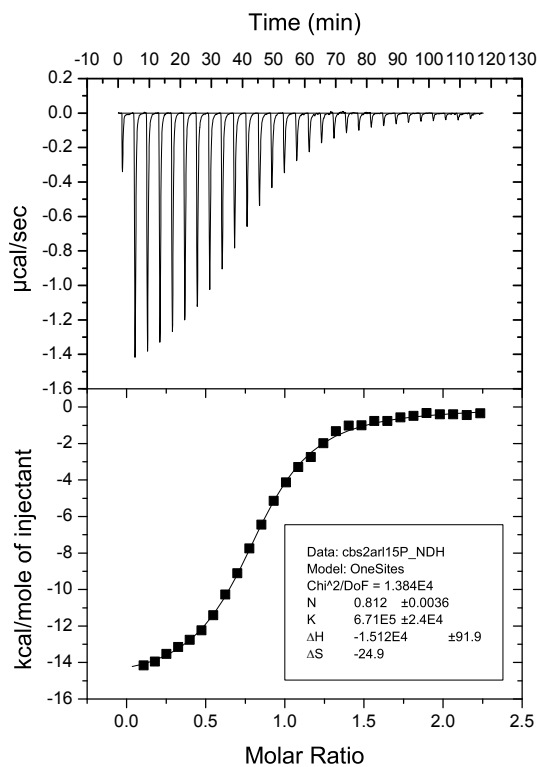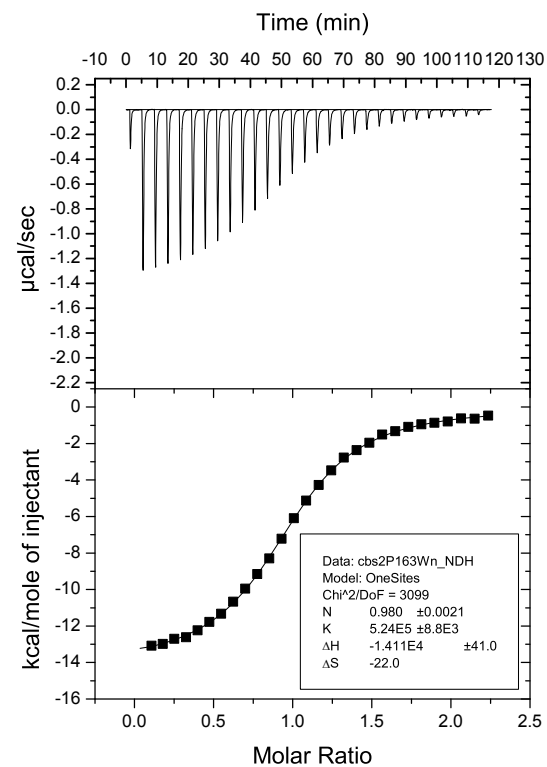

Supplement: Figure 4—source data 2. [file elife-86129-fig4-data2.pdf]

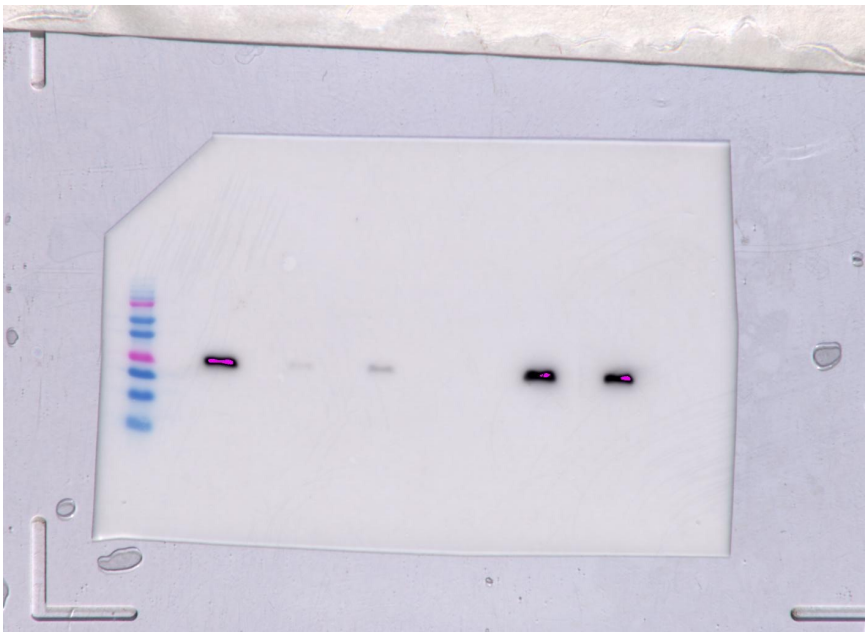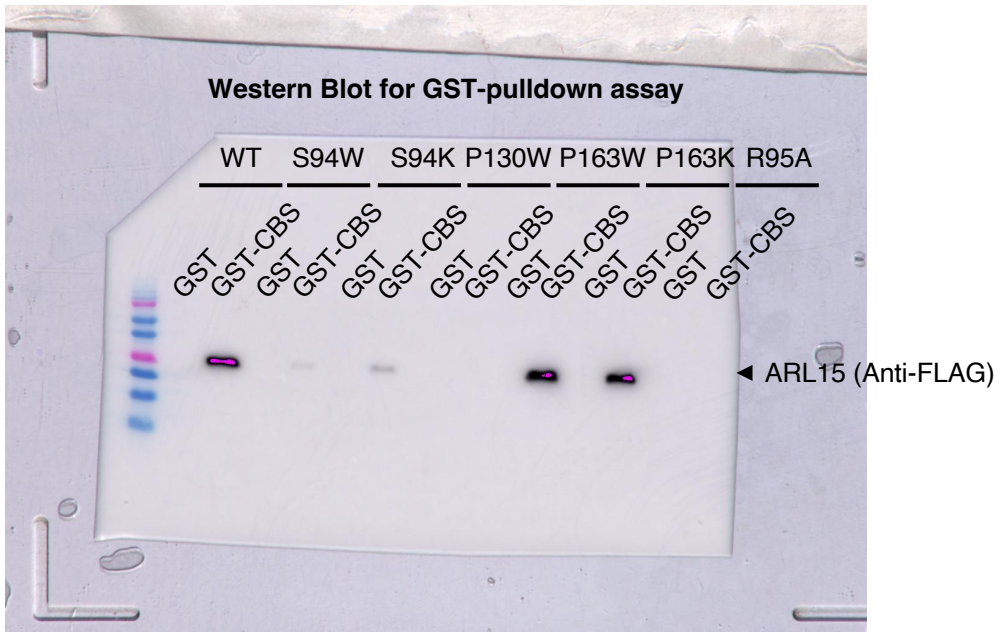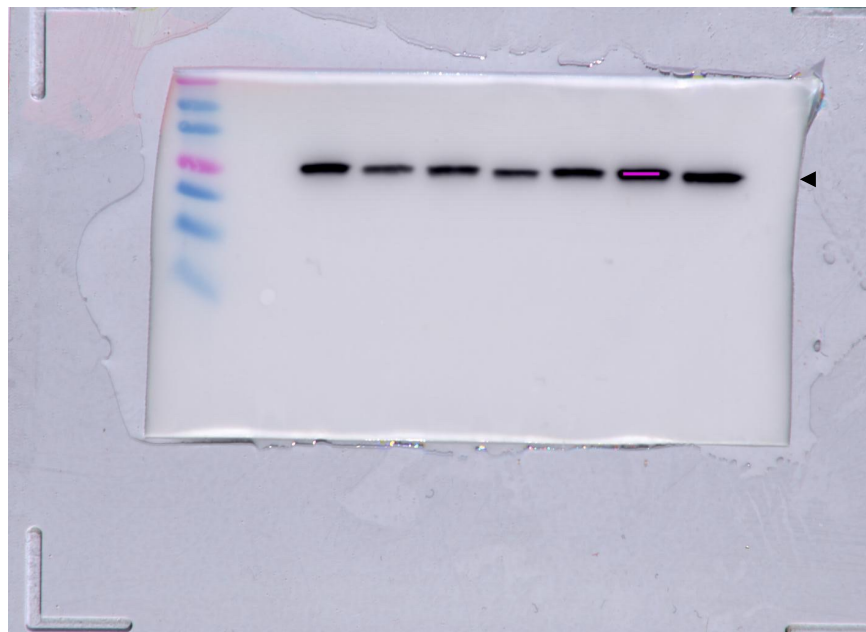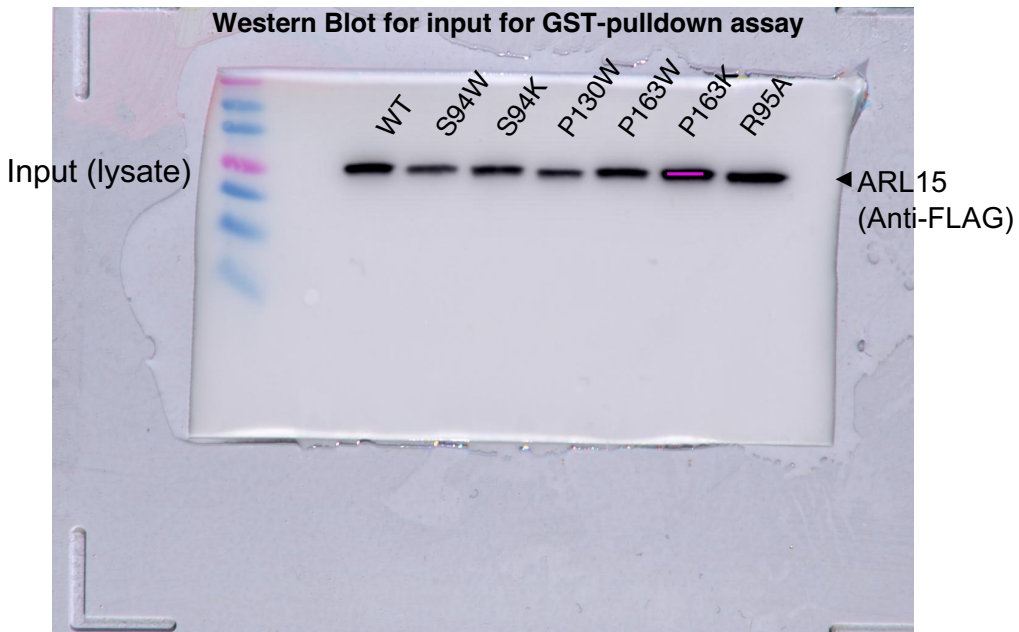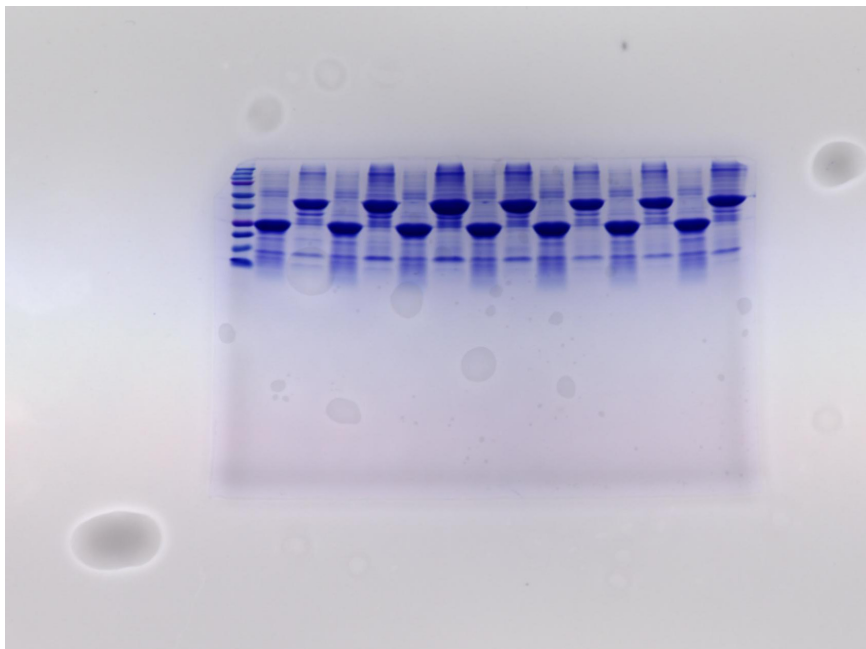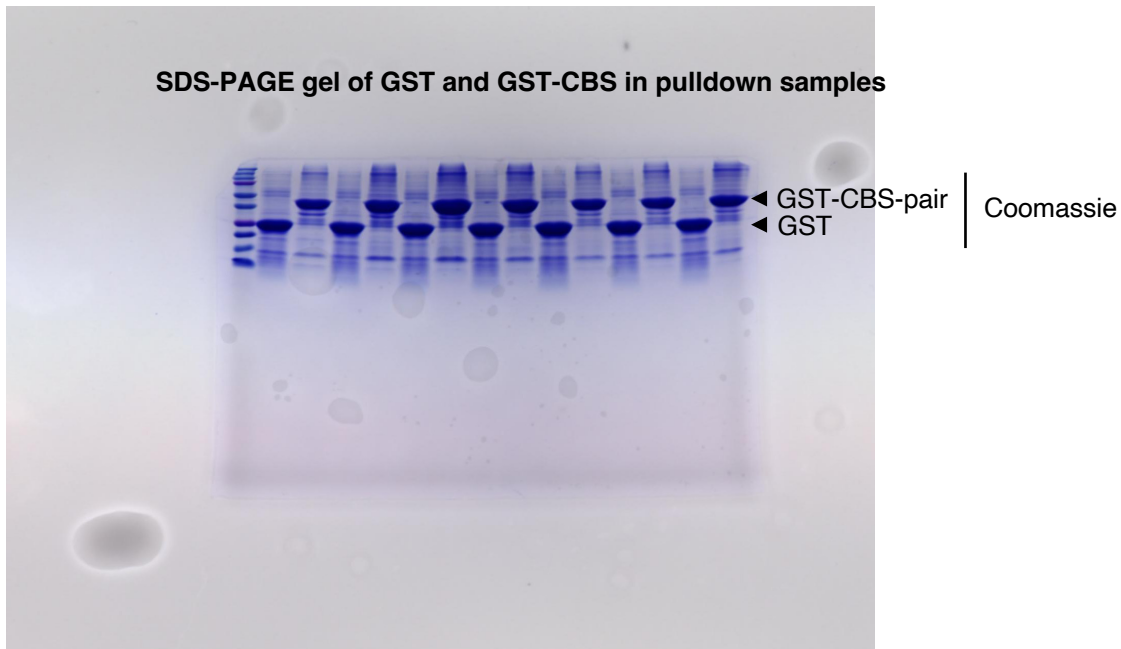

Supplement: Figure 4—source data 3. [file elife-86129-fig4-data3.pdf]

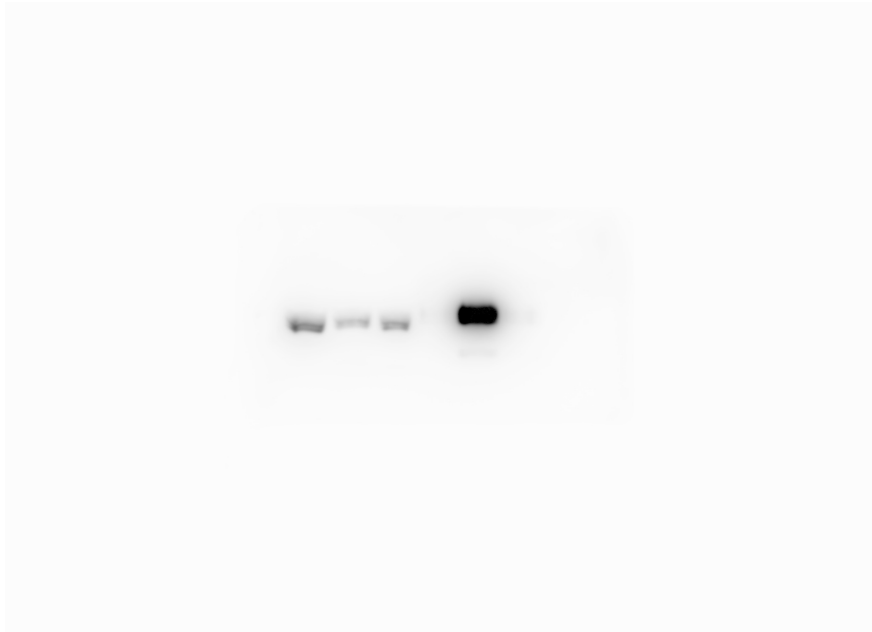

**Anti-CNNM3 Blot**

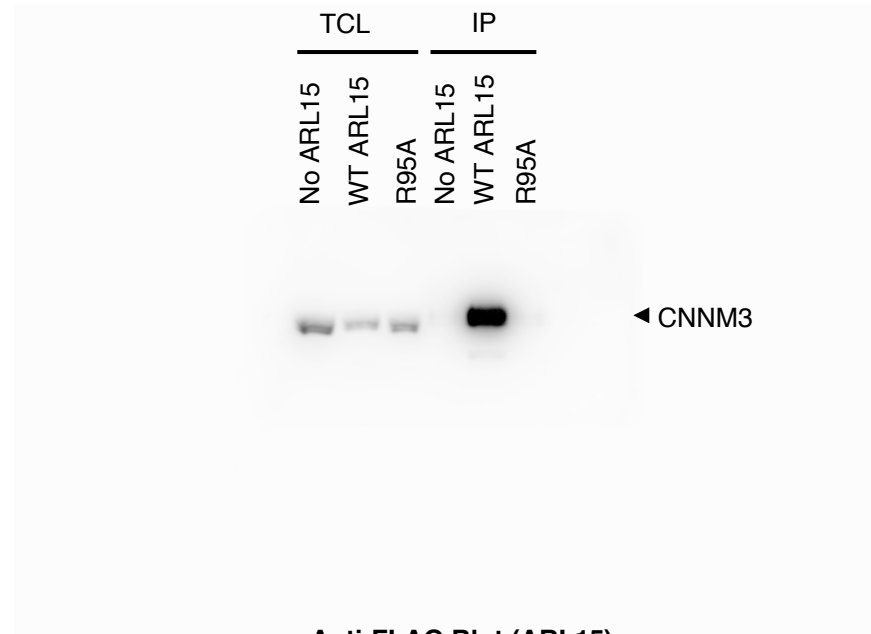

**Anti-FLAG Blot (ARL15)**

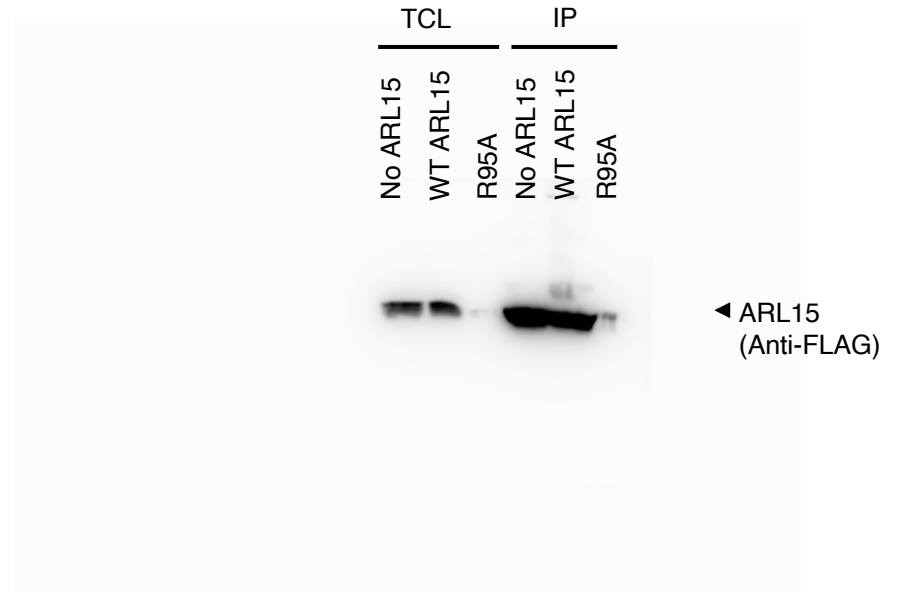

Supplement: Figure 4—source data 4. [file elife-86129-fig4-data4.pdf]

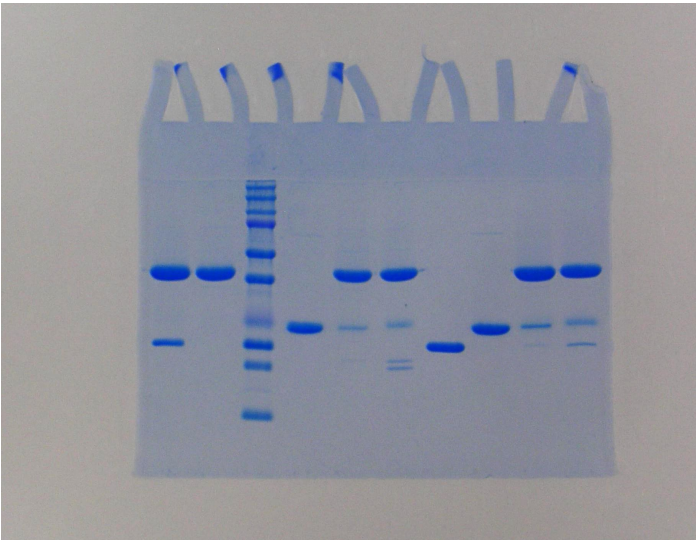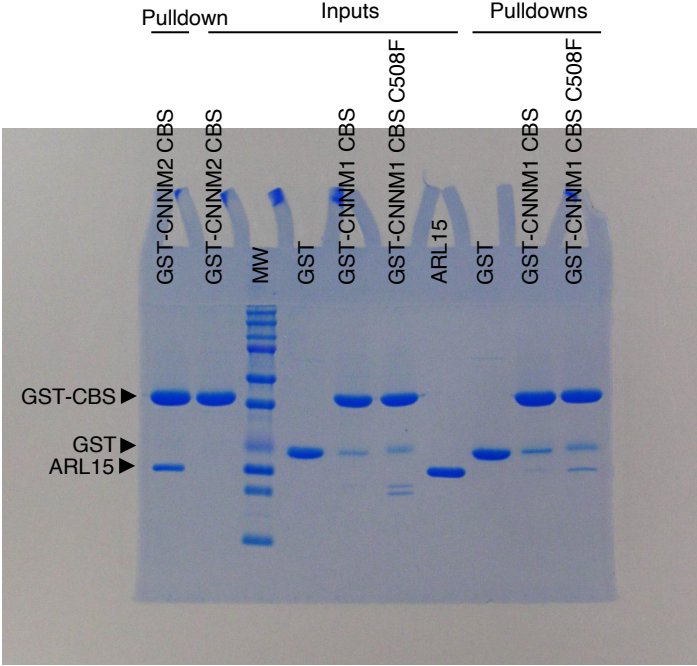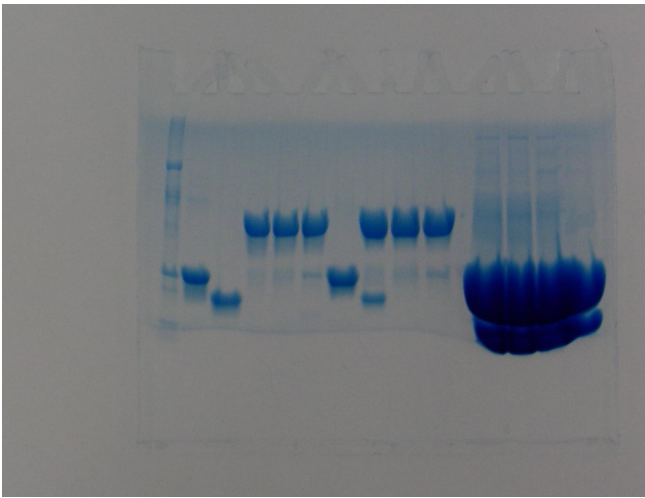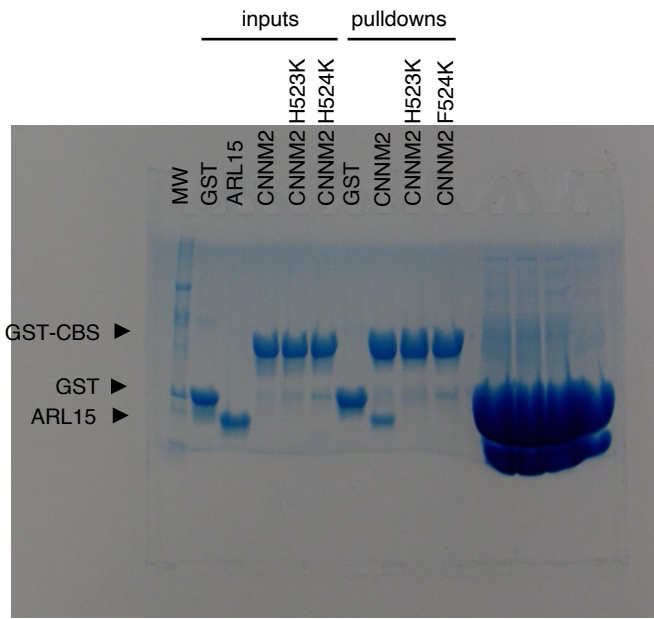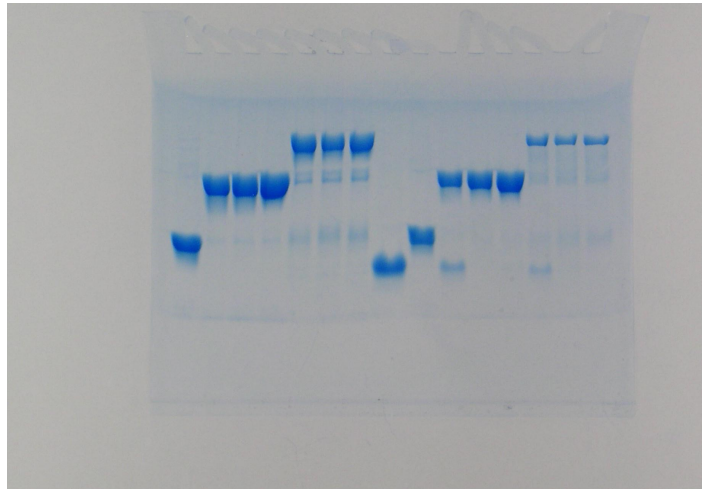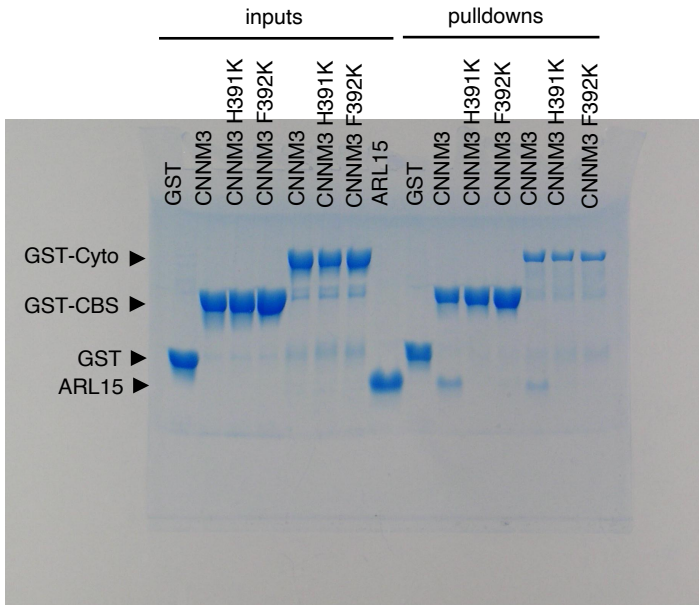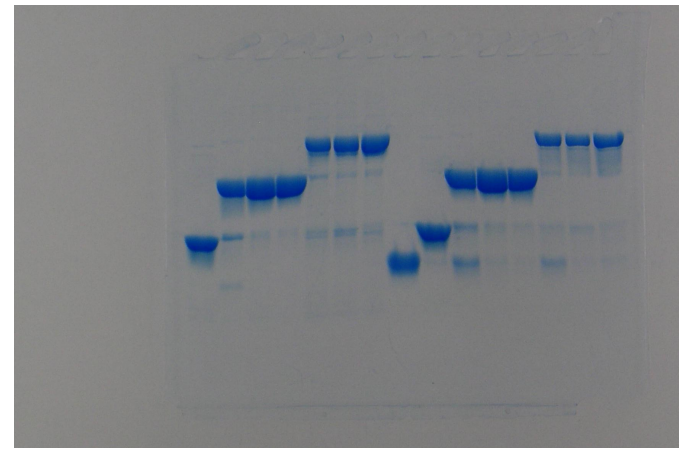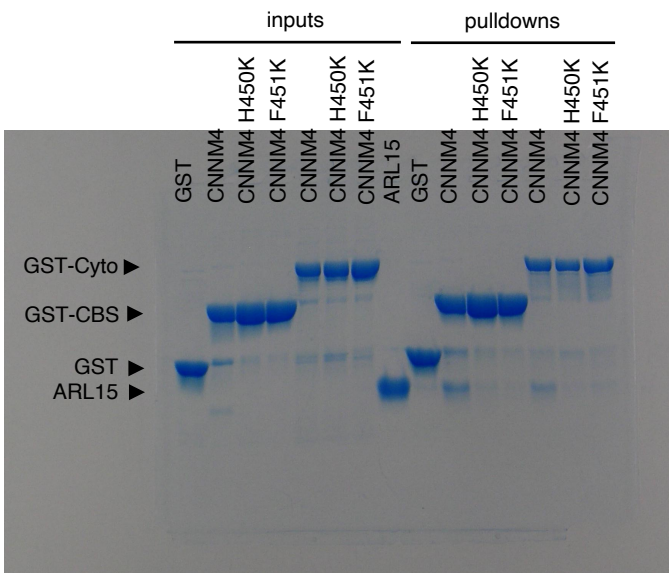

Supplement: Figure 5—source data 1. [file elife-86129-fig5-data1.pdf]

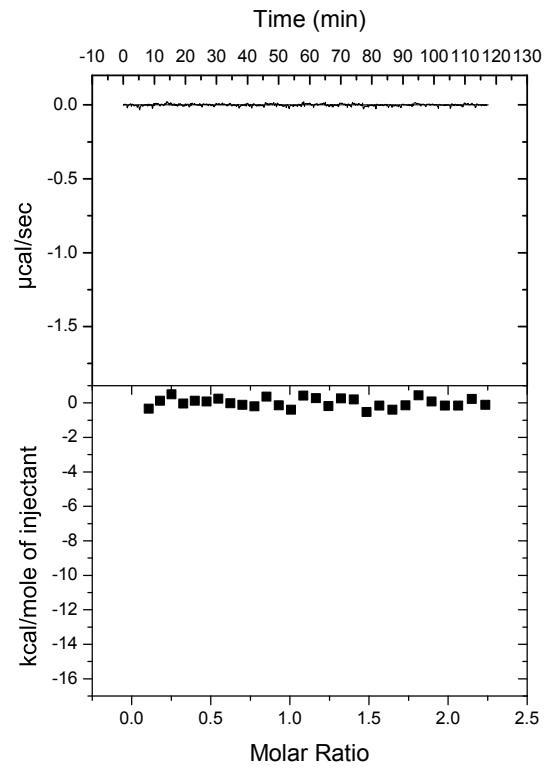

30  $\mu$ M CNNM2 429-584/45  $\mu$ M PRL2 (1-163)  
300  $\mu$ M ARL15 32-197

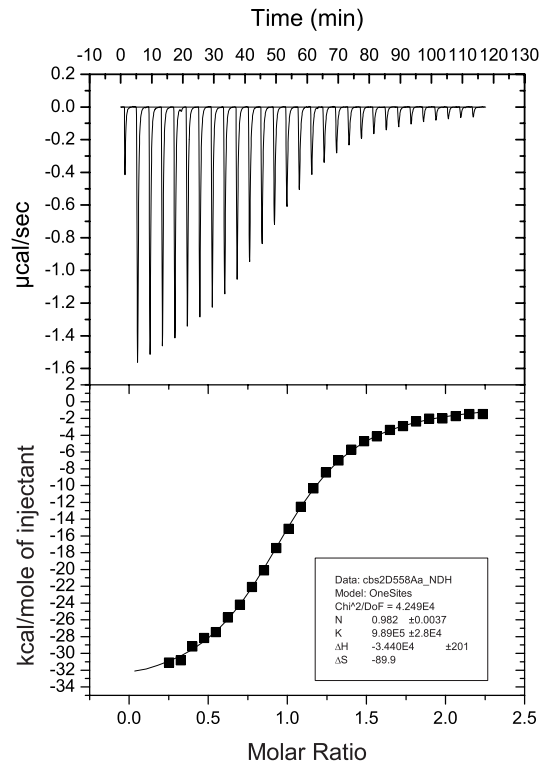

30  $\mu$ M CNNM2 429-584 **D558A**  
300  $\mu$ M ARL15 32-197

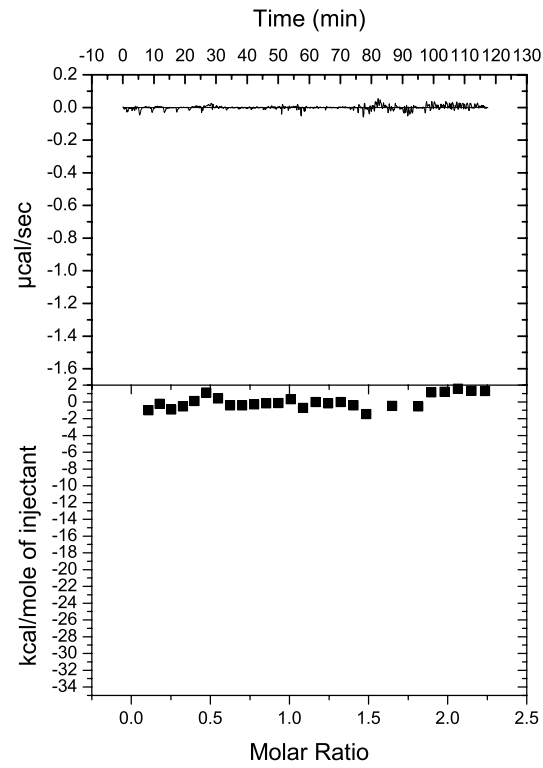

15  $\mu$ M CNNM2 429-584 **D558A**  
150  $\mu$ M PRL2 (1-163)

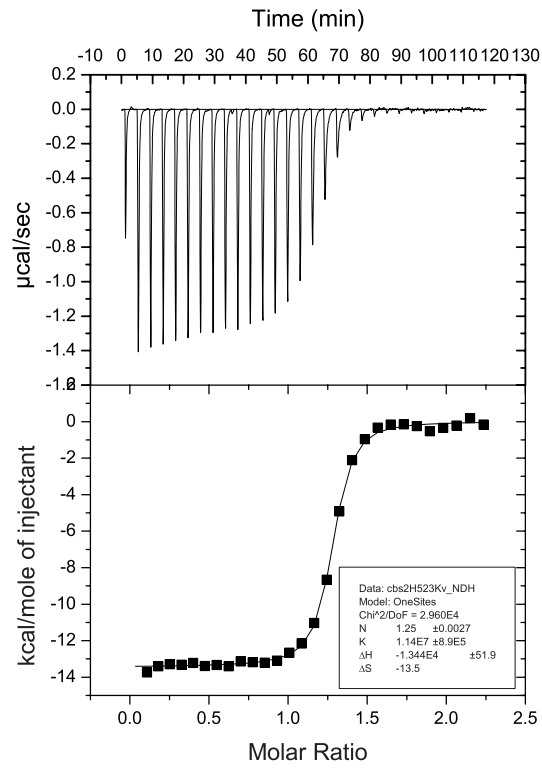

30  $\mu$ M CNNM2 429-584 **H523K**  
300  $\mu$ M PRL2 (1-163)

Supplement: Figure 6—source data 1. [file elife-86129-fig6-data1.pdf]

## Change in Mg Green Fluorescence

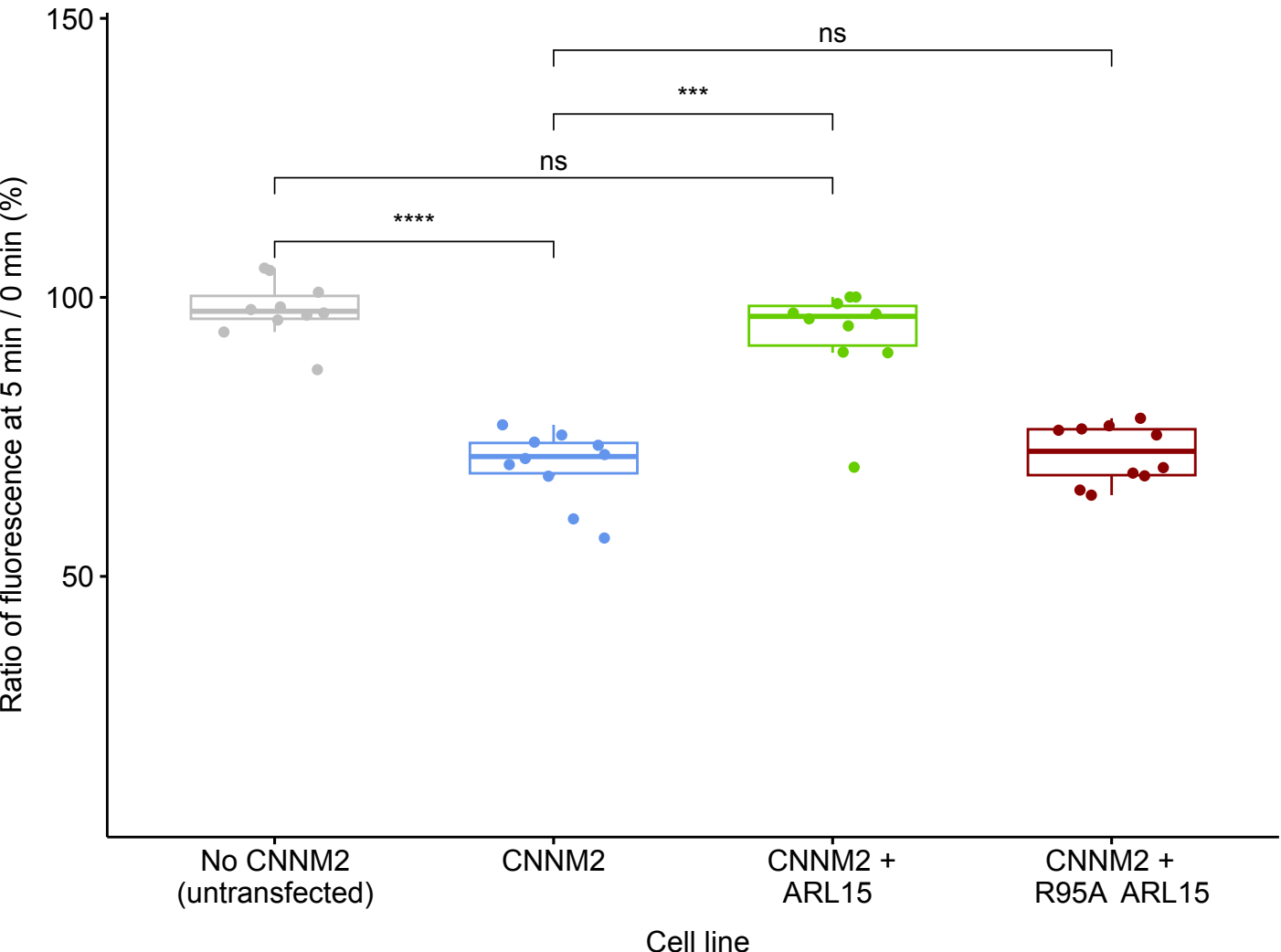

Supplement: Figure 7—source data 1. — The fluorescence for 10 cells in each condition was measured in the first and last frames of the 5 min efflux assay and the ratio calculated. The significance between conditions was evaluated via Wilcoxon analysis. The statistical analysis was performed in R suite, and the figure prepared using the ggplot2 extension package. [file elife-86129-fig7-data1.pdf]
