# Supplementary material for: Structural insights into regulation of CNNM-TRPM7 divalent cation uptake by the small GTPase ARL15
Source: eLife. 2023 Jul 14;12:e86129. doi: 10.7554/eLife.86129 (PMC10348743; doi:10.7554/eLife.86129)
Supplement: Figure 2—source data 1. [file elife-86129-fig2-data1.pdf]

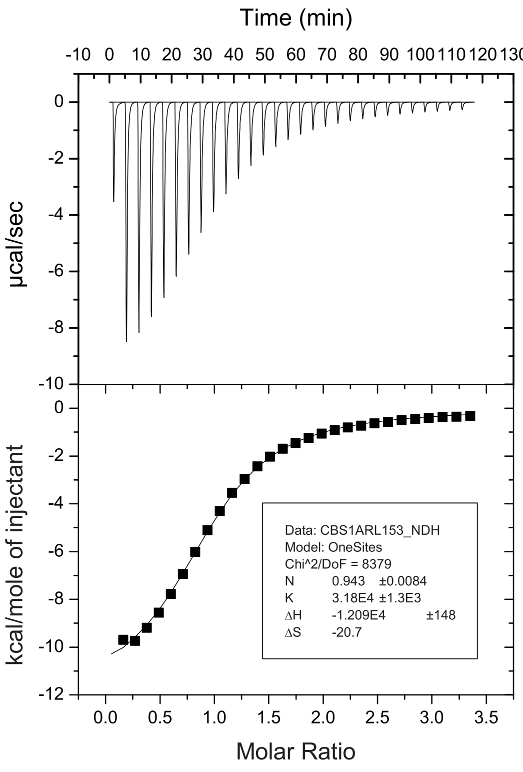

200 μM CNNM1 412-568
3 mM ARL15 32-197

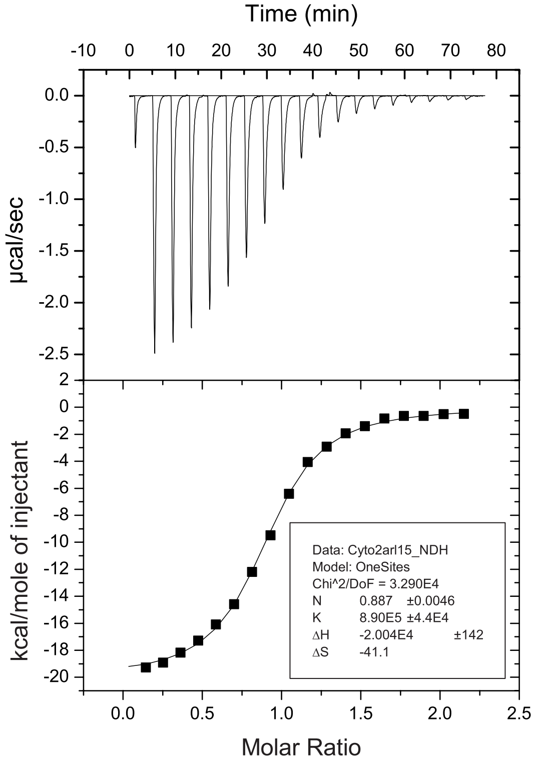

30 μM CNNM2 429-817
300 μM ARL15 32-197

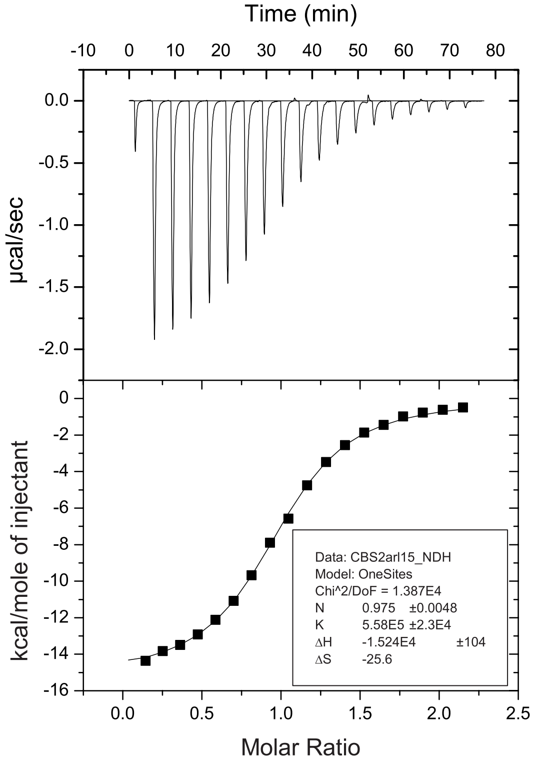

30 μM CNNM2 429-584
300 μM ARL15 32-197

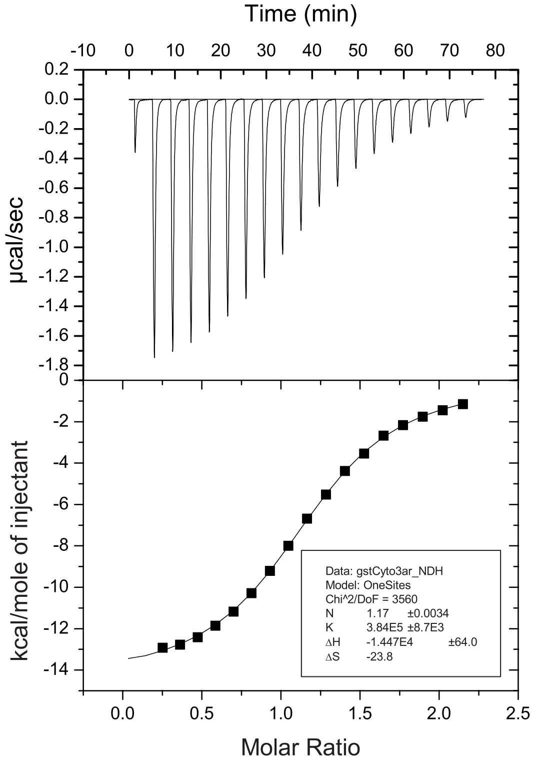

30 μM GST-CNNM3 299-658
300 μM ARL15 32-197

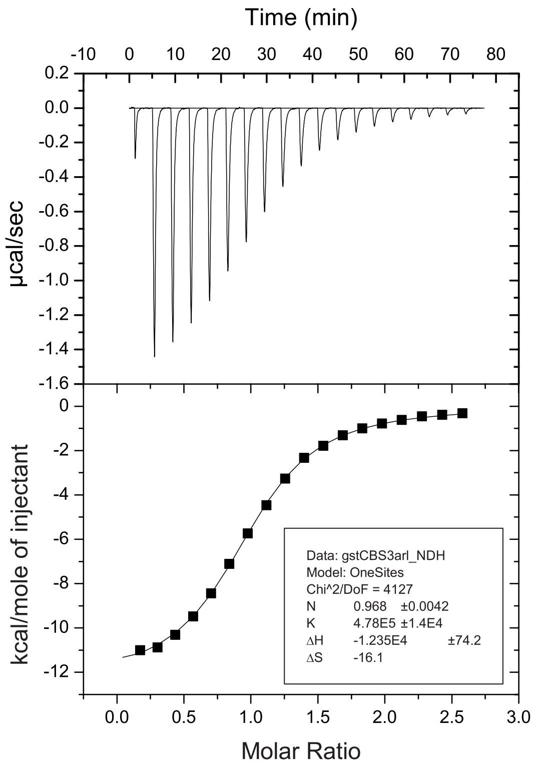

30 μM GST-CNNM3 299-452
300 μM ARL15 32-197

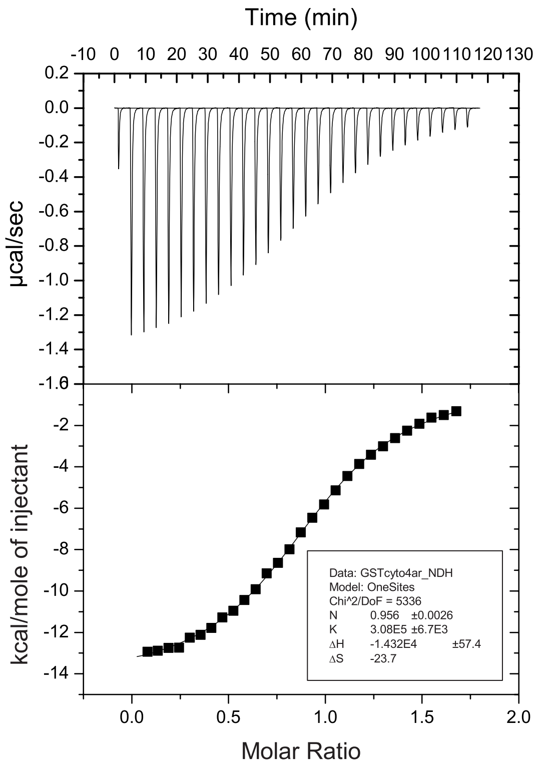

30 μM GST-CNNM4 356-726
300 μM ARL15 32-197

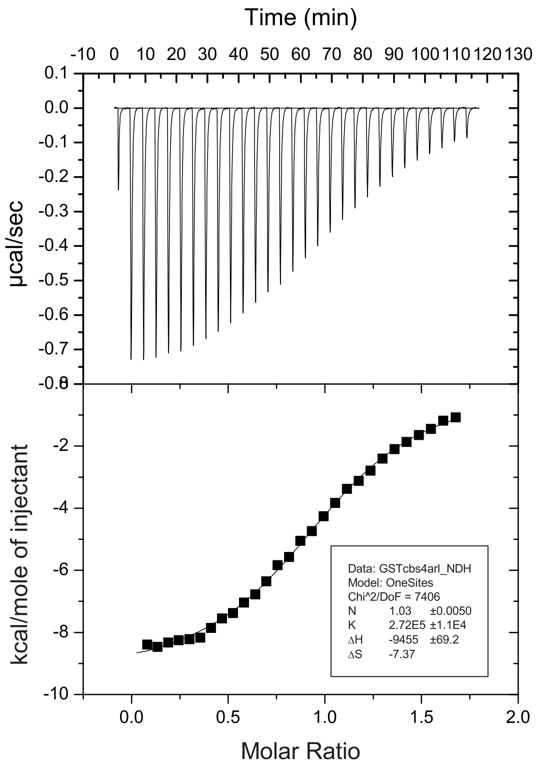

30 μM GST-CNNM4 356-511
300 μM ARL15 32-197
