# Supplementary material for: Structural insights into regulation of CNNM-TRPM7 divalent cation uptake by the small GTPase ARL15
Source: eLife. 2023 Jul 14;12:e86129. doi: 10.7554/eLife.86129 (PMC10348743; doi:10.7554/eLife.86129)
Supplement: Figure 2—source data 2. [file elife-86129-fig2-data2.pdf]

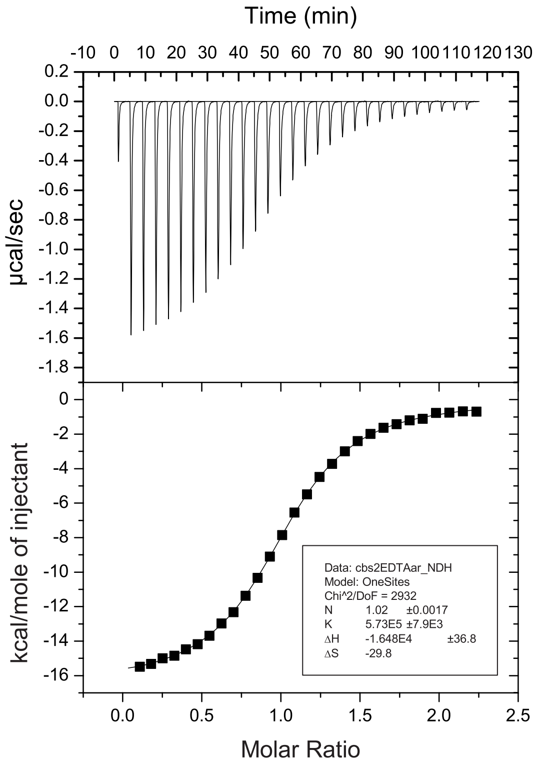

30 μM CNNM2 429-584
300 μM ARL15 32-197
in presence of 5 mM EDTA

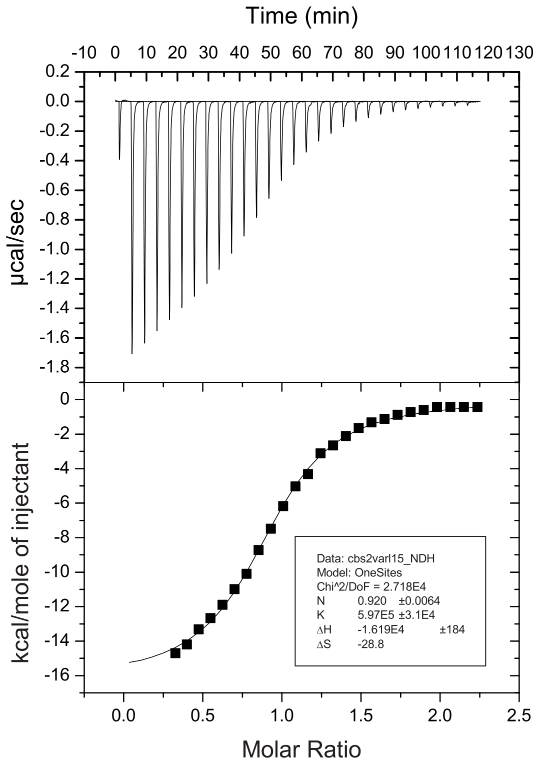

30 μM CNNM2 429-584
300 μM GTP-loaded ARL15 32-197
in presence of 1 mM $MgCl_2$

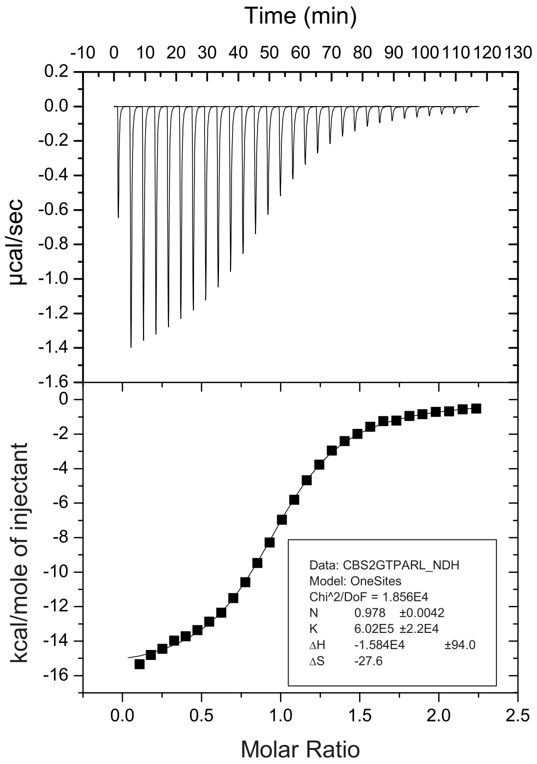

30 μM CNNM2 429-584
300 μM ARL15 32-197
in presence of 1 mM $MgCl_2$ and 1 mM GTP

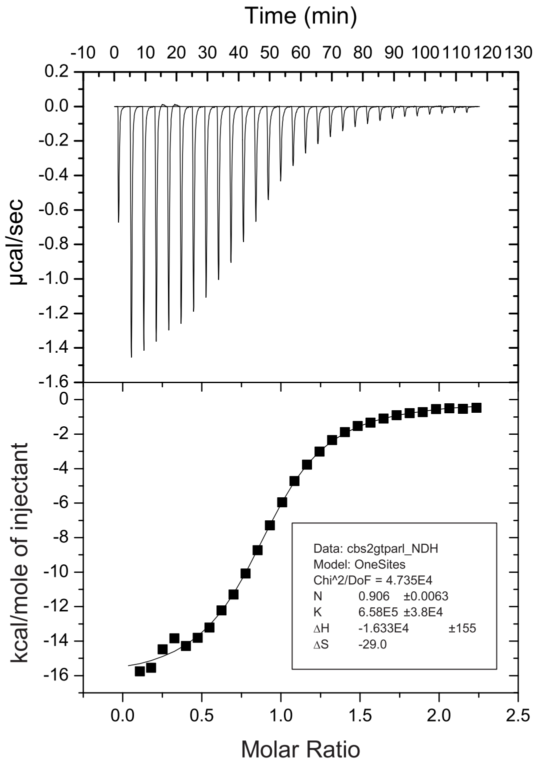

30 μM CNNM2 429-584
300 μM ARL15 32-197
in presence of 1 mM $MgCl_2$ and 1 mM GTP

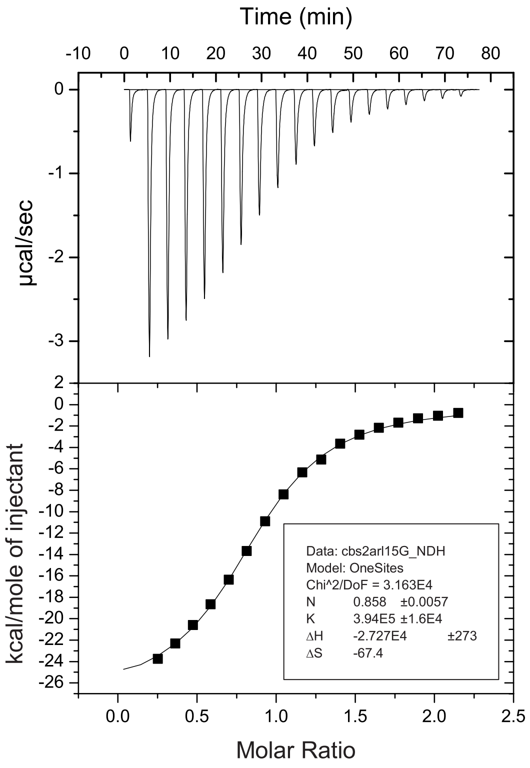

30 μM CNNM2 429-584
300 μM GDP-loaded ARL15 32-197
in presence of 1 mM $MgCl_2$

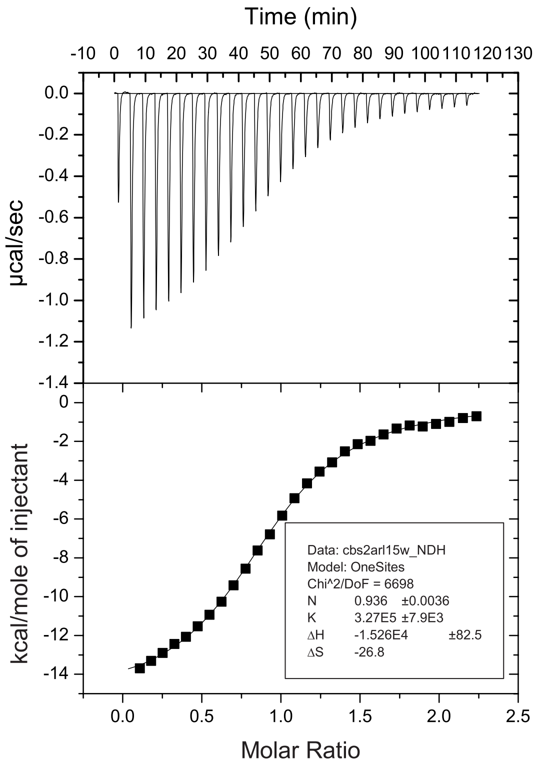

30 μM CNNM2 429-584
300 μM ARL15 32-197
in presence of 1 mM $MgCl_2$ and 3 mM GDP

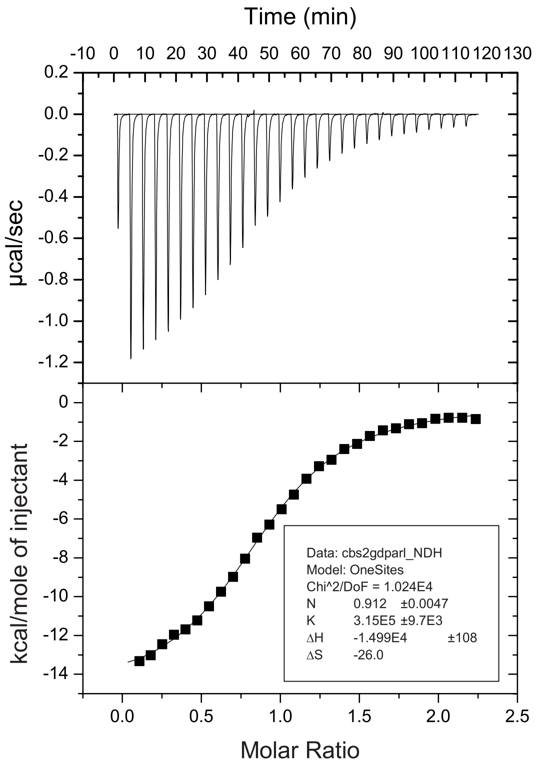

30 μM CNNM2 429-584
300 μM ARL15 32-197
in presence of 1 mM $MgCl_2$ and 3 mM GDP
