# Supplementary material for: Structural insights into regulation of CNNM-TRPM7 divalent cation uptake by the small GTPase ARL15
Source: eLife. 2023 Jul 14;12:e86129. doi: 10.7554/eLife.86129 (PMC10348743; doi:10.7554/eLife.86129)
Supplement: Figure 2—source data 3. [file elife-86129-fig2-data3.pdf]

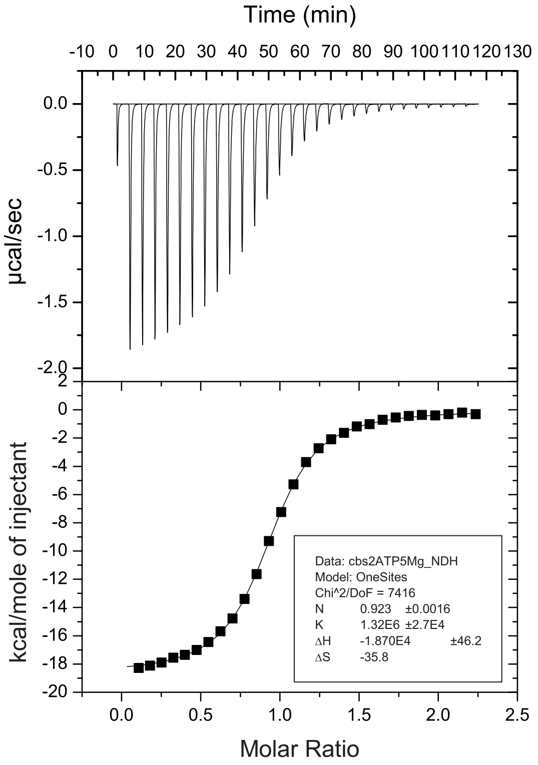

30 μM CNNM2 429-584
300 μM ARL15 32-197
in presence of 1 mM ATP
and 5 mM $MgCl_2$

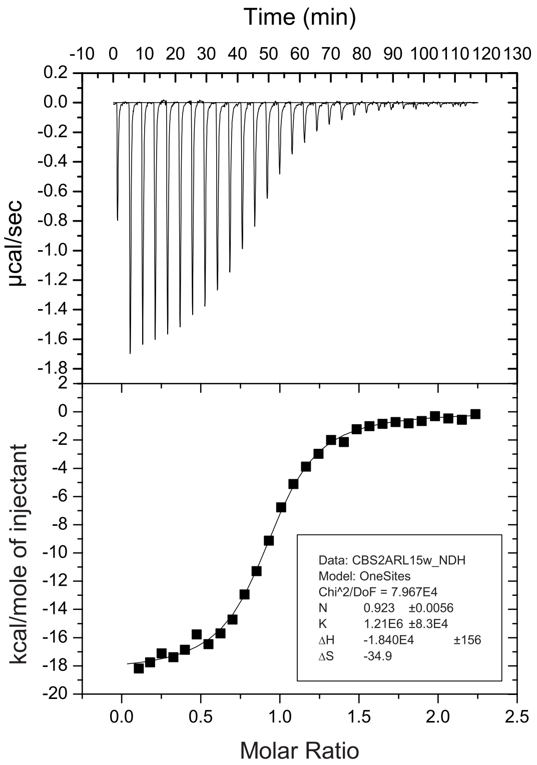

30 μM CNNM2 429-584
300 μM ARL15 32-197
in presence of 1 mM ATP
and 5 mM $MgCl_2$

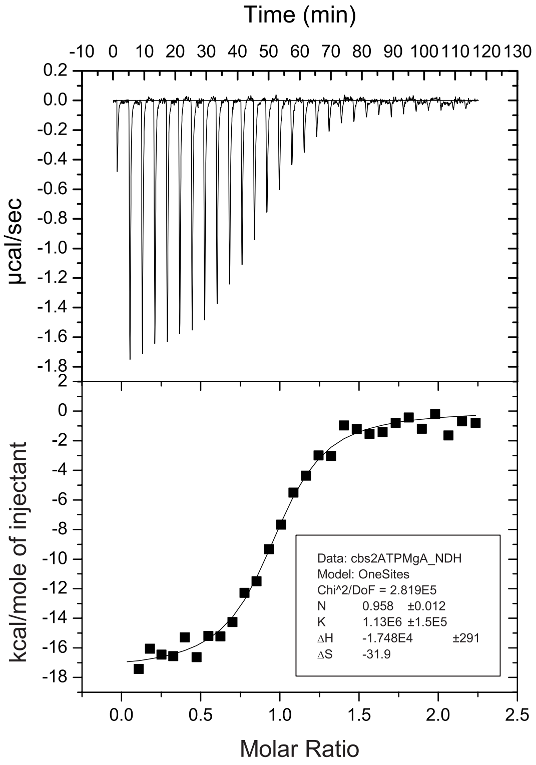

30 μM CNNM2 429-584
300 μM ARL15 32-197
in presence of 1 mM ATP
and 50 mM $MgCl_2$

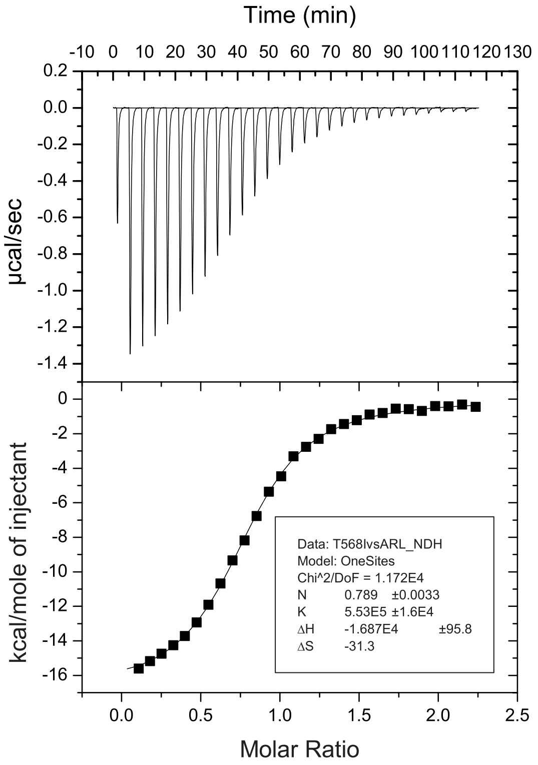

30 μM CNNM2 429-584 **T568I**
300 μM ARL15 32-197

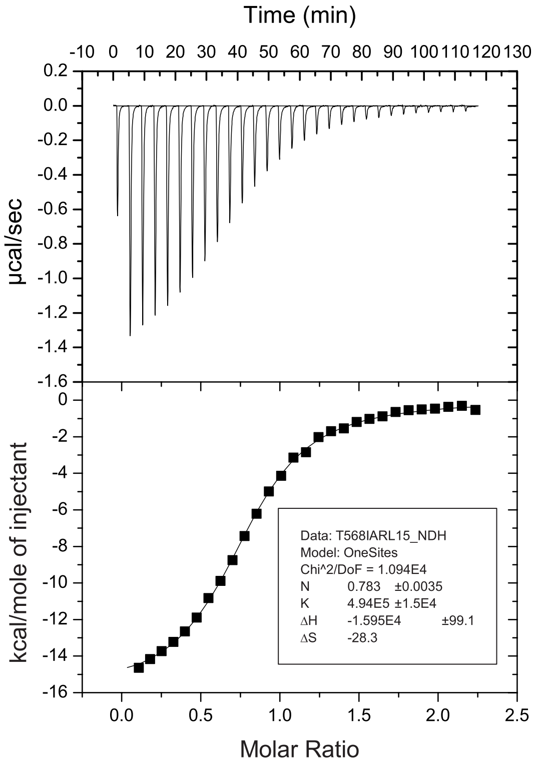

30 μM CNNM2 429-584 **T568I**
300 μM ARL15 32-197
in presence of 1 mM ATP
and 5 mM $MgCl_2$
