# Supplementary material for: Structural insights into regulation of CNNM-TRPM7 divalent cation uptake by the small GTPase ARL15
Source: eLife. 2023 Jul 14;12:e86129. doi: 10.7554/eLife.86129 (PMC10348743; doi:10.7554/eLife.86129)
Supplement: Figure 5—source data 2. [file elife-86129-fig5-data2.pdf]

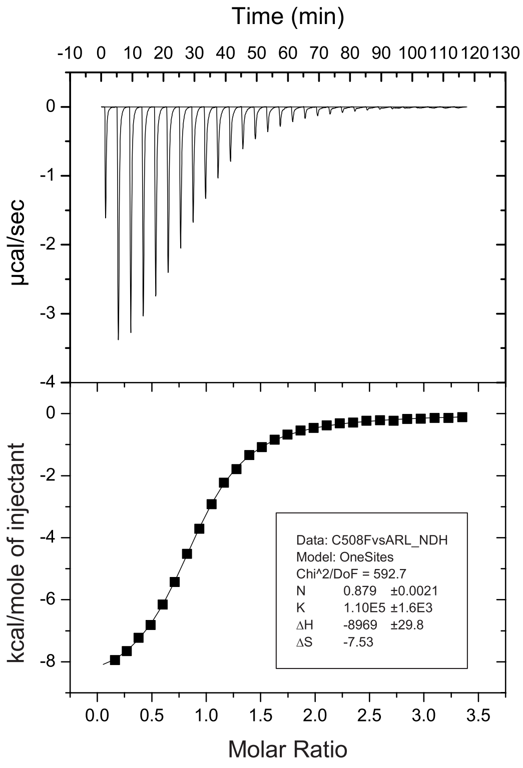

100 μM CNNM1 412-568 **C508F**
1.5 mM ARL15 32-197

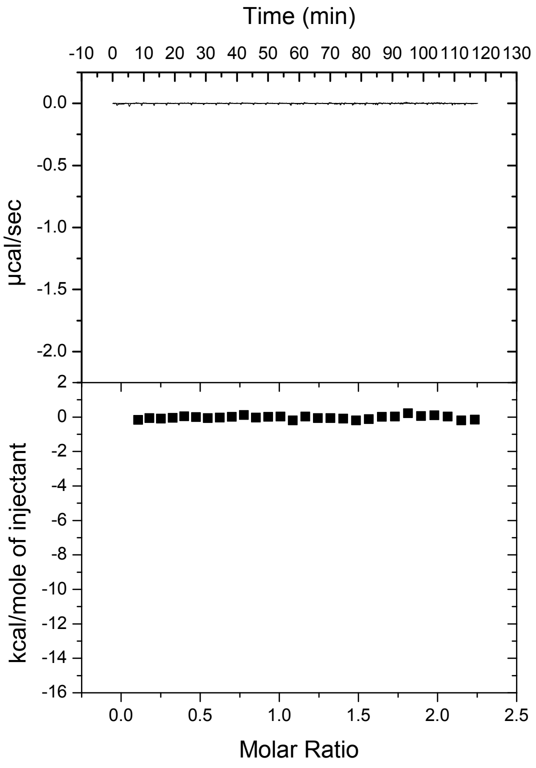

30 μM CNNM2 429-584 **H523K**
300 μM ARL15 32-197

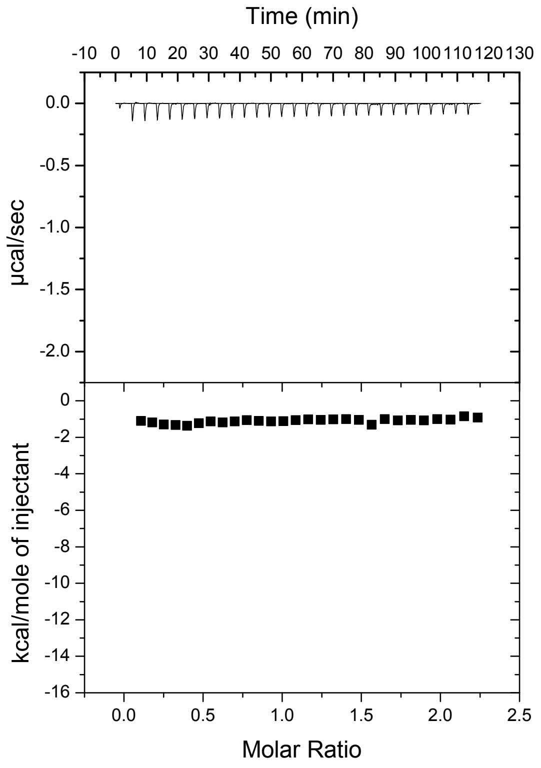

30 μM CNNM2 429-584 **F524K**
300 μM ARL15 32-197

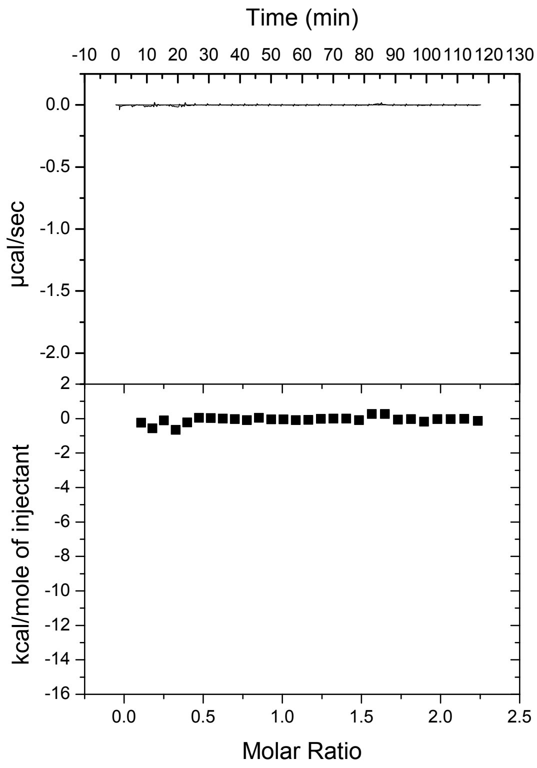

30 μM CNNM3 299-452 **H391K**
300 μM ARL15 32-197

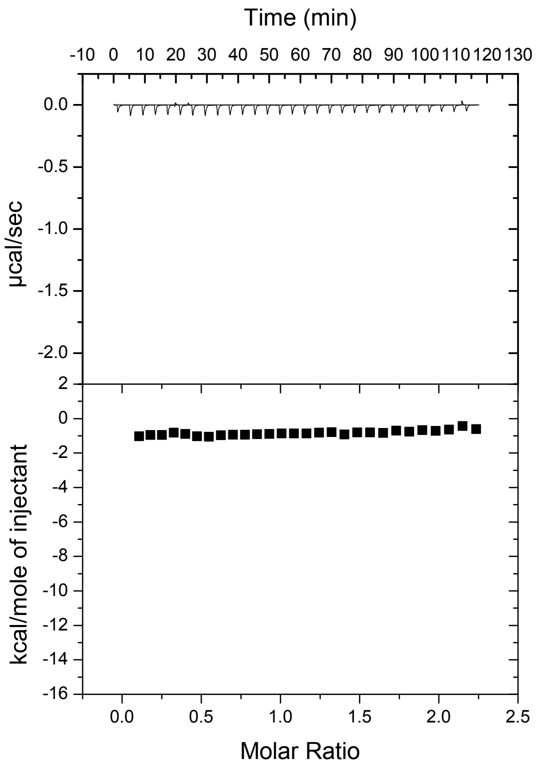

30 μM CNNM3 299-452 **F392K**
300 μM ARL15 32-197

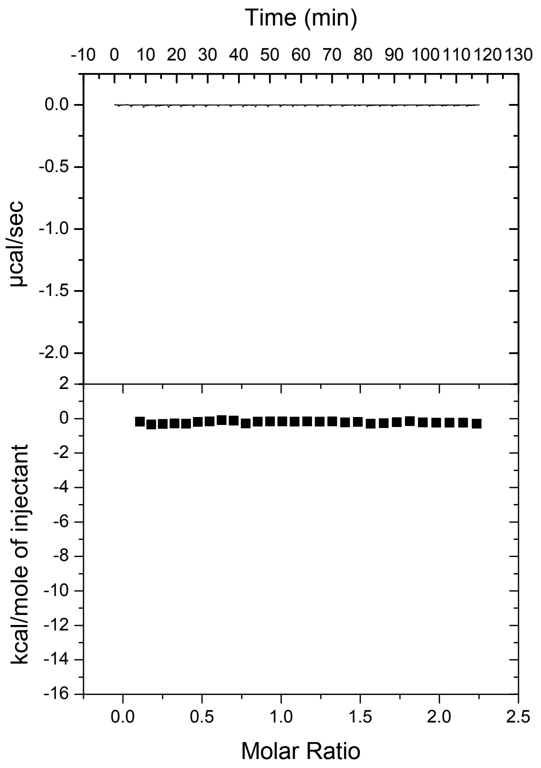

30 μM CNNM4 356-511 **H450K**
300 μM ARL15 32-197

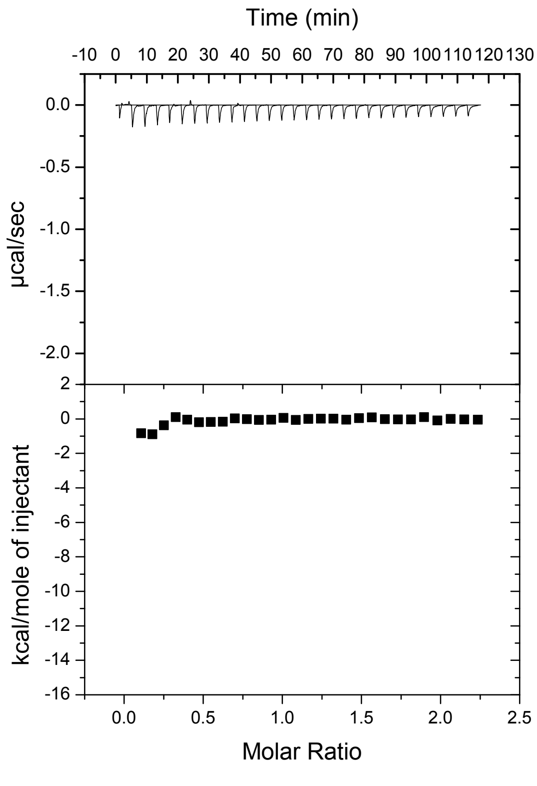

30 μM CNNM4 356-511 **F451K**
300 μM ARL15 32-197

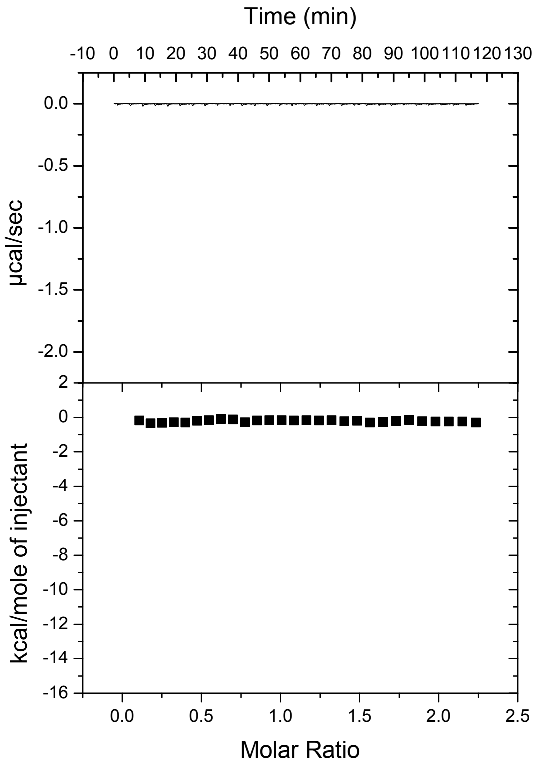

30 μM CNNM4 356-726 **H450K**
300 μM ARL15 32-197

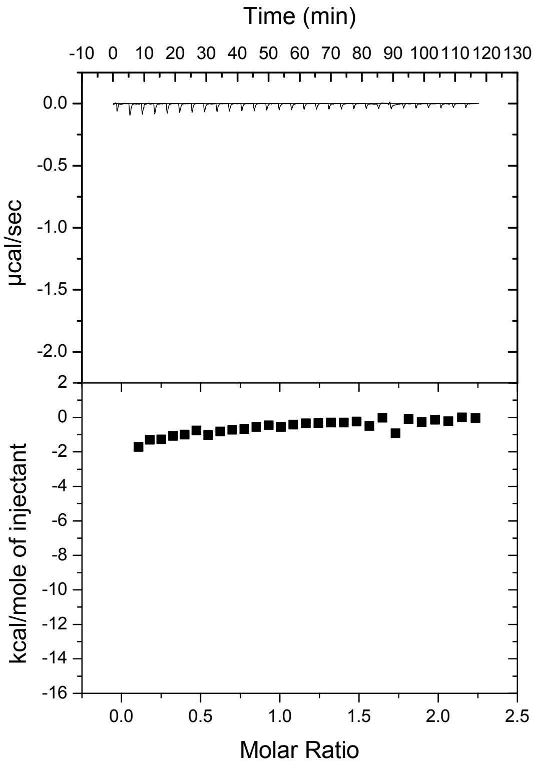

30 μM CNNM4 356-726 **F451K**
300 μM ARL15 32-197
